# A Self-Referenced Diffraction-Based Optical Leaky Waveguide Biosensor Using Photofunctionalised Hydrogels

**DOI:** 10.3390/bios10100134

**Published:** 2020-09-24

**Authors:** Anil K. Pal, Nicholas J. Goddard, Hazel J. Dixon, Ruchi Gupta

**Affiliations:** 1 School of Chemistry, University of Birmingham, Birmingham B15 2TT, UK; a.k.pal@bham.ac.uk (A.K.P.); hjd839@student.bham.ac.uk (H.J.D.); 2 Process Instruments (UK) Ltd., Burnley BB12 0BT, UK

**Keywords:** self-referenced, leaky waveguide, biosensor, hydrogel, photocleavable

## Abstract

We report a novel self-referenced diffraction-based leaky waveguide (LW) comprising a thin (~2 µm) film of a photofunctionalisable hydrogel created by covalent attachment of a biotinylated photocleavable linker to chitosan. Streptavidin attached to the chitosan via the photocleavable linker was selectively removed by shining 365 nm light through a photomask to create an array of strips with high and low loading of the protein, which served as sensor and reference regions respectively. The differential measurements between sensor and reference regions were used for measuring analytes (i.e., biotin protein A and IgG) while reducing environmental and non-specific effects. These include changes in temperature and sample composition caused by non-adsorbing and adsorbing species, leading to reduction in effects by ~98%, ~99%, and ~97% respectively compared to the absolute measurements. The novelty of this work lies in combining photofunctionalisable hydrogels with diffraction-based LWs for referencing. This is needed to realise the full potential of label-free optical biosensors to measure analyte concentrations in real samples that are complex mixtures, and to allow for sample analysis outside of laboratories where drifts and fluctuations in temperature are observed.

## 1. Introduction

Leaky waveguides (LWs) are a type of label-free optical biosensor, comprising of a few microns thick film with refractive index (RI) lower than the substrate but higher than the sample applied to the sensor [1,2,3]. LWs have been used for sensing different types of analytes including vapours [4], small molecules in solutions [5,6], microbes [7], and proteins [8,9,10,11]. In 2020, we observed a novel manifestation in LWs with low RI difference (0.002–0.005 RIU) between the waveguide and sample, and reported its suitability for the direct visualisation of the resonance angles as exponentially decaying interference fringes [12]. These LWs are called diffraction-based LWs [13] and offered ~13 times higher figure of merit (FOM), which is the ratio of the refractive index sensitivity (RIS) and full width half maximum (FWHM) of the waveguide resonance, than surface plasmon resonance (SPR). Generally speaking, the higher the FOM, the easier it is to determine small shifts in the resonance angle observed at low analyte concentrations. Other benefits of LWs include ease of integration with electric fields for in-situ sample pre-concentration allowing for rapid analysis at low analyte concentrations [14], and fabrication using completely solution-processed methods and widely available materials for production of affordable biosensors. A limitation of the LWs is that their RIS is determined by the porosity of the waveguide layer to the analyte, and making mesoporous films with a thickness of a few microns is challenging [13].

The position of the resonance angle is a function of RI of the waveguide and the solution in its evanescent field. In addition to binding of analytes to recognition elements (REs) immobilised in LWs, the RI can also change because of common-mode effects i.e., variations in temperature, wavelength of light sources and sample composition. The temperature coefficient of RI of an aqueous solvent is ~0.9 × 10^−4^ RIU/°C at room temperature [15,16], often resulting in much higher RI changes than that caused by analyte binding unless rigorous temperature control is applied. Temperature changes can also cause the peak wavelength of light sources to drift and “mode hops” in laser diodes [17]. Equally, the concentration of analytes is often several orders of magnitude lower than interferents in samples. The effect of changes in sample composition can be alleviated by integrating sample processing with biosensors for the removal of interferents and preconcentration of analytes. For example, electrokinetic sample processing has been integrated with LWs [14], but cannot compensate for changes in RI caused by other common-mode effects.

The contributions of analyte binding and common-mode effects can be separated by referencing [18,19,20] where typically differential measurements between the outputs of the sensor and reference regions are performed. Commercial instruments such as BIACORE have multiple fluidic channels where at least one of the channels is used for referencing [18]. The use of multiple fluidic channels often results in solutions reaching the sensor and reference channels at slightly different times, resulting in spikes in the differential signal. The presence of these spikes often makes it difficult to study the binding kinetics between the analyte and REs as well as to extract their association and dissociation constants. Sensor and reference regions have also been fabricated in a single fluidic channel by selective immobilisation of REs, which is typically achieved via microcontact printing [19,20,21]. Alternatively, electrolytic grafting of diazonium salts for selective immobilisation of REs in closed channel format has been reported for making referenced SPR biosensors [22]. The number of sensor and reference spots that can be fabricated using the approach is, however, determined by the number of available electrodes. In comparison to pre-fabricated electrodes, the use of light combined with digital photomasks is beneficial because the shape and distribution of sensor and reference regions can be rapidly changed on-demand. Towards this end, photocleavable linkers for patterning biomolecules on surfaces [23] and hydrogels [24] have been reported. The photolysis kinetics of these photocleavable linkers has been studied using label-free detection methods [23], but their application for the fabrication of sensor and reference regions of label-free optical biosensors has so far not been reported.

This is the first report of a diffraction-based LW with an array of alternate strips of different RI where the resonance angle corresponding to each strip was independently visualised as interference fringes. The ability to pattern low index hydrogels opens up the possibility of self-referencing and multiplexing while still retaining the high FOM of diffraction-based LW sensors. The strips were ~1 mm wide and were fabricated by activating a chitosan waveguide with a photocleavable (PC) biotin linker, which was then allowed to bind to streptavidin. Subsequent photofunctionalisation by exposure to 365 nm light through a photomask (a Ronchi grating) selectively removed the linker and streptavidin. This work also demonstrates that the alternate strips with high and low loading of streptavidin can serve as sensor and reference regions. We showed that the differential response between these sensor and reference regions was suitable for analyte (in this work, IgG) sensing while reducing the impact of undesirable side effects. The effect of changes in temperature over 5 °C and sample composition because of non-adsorbing and adsorbing species were reduced by ~98%, ~99%, and ~97% respectively compared to the absolute shifts in the resonance angles. Our theoretical modelling also shows that diffraction-based LWs are significantly less dispersive [25] than other optical sensors (e.g., SPR [26] and resonant mirror (RM) [27]) and hence are less susceptible to variations in the wavelength of light sources. We can define a dispersion figure of merit (DFOM), which is the angular FWHM of the resonance peak or dip divided by the sensitivity of the resonance angle to wavelength, and has the units of wavelength. It is the wavelength change needed to move the resonance angle by the FWHM of the resonance. Table 1 gives the theoretical DFOM for SPR, RM and diffraction-based LWs operating at 650 nm obtained using transfer matrix modelling. It can be seen that the diffraction-based LWs, particularly when using TE polarised light, have the best DFOM, while the RM using TE polarised light provides the worst DFOM.

## 2. Experimental

### 2.1. Materials

1 M acetic acid, 1 M sodium hydroxide, methanol, acetone, 25% (*v*/*v*) glutaraldehyde (GA), glycerol (M_w_: 92), poly(ethylene glycol) (PEG) (M_w_: 10, 100 and 300 kD), protein A–biotin (PAB) from Staphylococcus aureus (P2165), IgG from sheep serum (I5131), semicarbazide hydrochloride (ScZ), sodium phosphate monobasic monohydrate, sodium phosphate dibasic dodecahydrate, bovine serum albumin (BSA), and anhydrous dimethyl sulfoxide (DMSO) were purchased from Sigma Aldrich (UK). HEPES and decon 90 were purchased from Fisher Scientific (UK). Streptavidin (2-0203-100) was purchased from IBA Lifesciences (Germany). PC Biotin-PEG_3_-NHS carbonate ester (PC-biotin) with purity of ~95% was purchased from BroadPharm (USA). The above-purchased chemicals were used as received without further purification. Chitosan (M_w_: 100–300 kD, 90% deacetylated) was purchased from Sigma Aldrich (UK) and purified by following the steps described in our previous report [28]. Glass microscope slides were purchased from VWR (UK), dialysis tubing cellulose membrane (molecular weight cut-off: 14,000) was purchased from Sigma Aldrich (UK), and syringe filters with cut-off size of 5 µm were purchased from Scientific Lab Suppliers (UK). 100 mM phosphate buffer (PB) solution at pH 8.5 was prepared by dissolving appropriate amounts of sodium phosphate monobasic and dibasic salts in de-ionised (DI) water. 100 mM HEPES buffer solution of pH 7.4 was also prepared.

### 2.2. Fabrication of Diffraction-Based LWs and Their Photopatterning

The glass substrates of ~25 × 25 mm^2^ were cleaned in decon 90 solution, DI water, and ethanol by sonication for 30 min each, and then dried in a hot air oven operated at 60 °C. Finally, the substrates were cleaned using a plasma cleaner (Harrick Plasma, PDC-002-CE).

The purified chitosan was dissolved in 0.1 M acetic acid under continuous stirring for 18 h to form a 1% (*w*/*v*) solution [28]. 150 µL of chitosan solution was spin coated onto the glass substrate at a spin speed of 900 rpm for 30 s with acceleration of 100 rpm/s inside a laminar flow cabinet. The spin coated substrates were placed inside an incubator maintained at 25 °C and humidity of 75–80% for 3 min. The chitosan films were then crosslinked by immersing them in 0.03% (*v*/*v*) GA prepared in HEPES buffer for 5 min, washed with HEPES buffer to remove unreacted GA, and stored in HEPES buffer until further use.

Chitosan films were treated with 500 µL of PC-biotin of different concentrations for 40 min and then washed with HEPES buffer. Subsequently, the films were treated with streptavidin (0.05 mg/mL) for 40 min and washed with HEPES buffer. Finally, the chitosan films with immobilised streptavidin were immersed in 1.25% (*w*/*v*) ScZ prepared in HEPES buffer. The light emitted by a 365 nm LED (Nichia NVSU233A-U365, 1030 mW) was collimated by passing through a 25 mm diameter planoconvex lens, resulting in a beam with a power density of ~210 mW cm^−2^. The collimated beam was passed through a Ronchi grating of pitch 1 mm, and the resulting pattern of bright and dark lines was projected perpendicularly on the chitosan films for different durations.

### 2.3. Preparation of Solutions for Characterisation of LWs

Glycerol and different PEG (M_w_: 10, 100 and 300 kD) solutions were prepared in HEPES buffer and used to estimate the porosity of chitosan LWs. The received lyophilised powder of PC-biotin was dissolved in anhydrous DMSO in an inert atmosphere (N_2_ gas) at 10 mg/mL and stored in a freezer in darkness for further use. The aliquots were diluted in HEPES buffer before use to obtain PC-Biotin solutions of different concentrations. BSA, streptavidin, PAB, and IgG solutions were prepared in HEPES buffer with concentrations of 1, 0.05, 0.07, and 0.1 mg/mL, respectively.

### 2.4. Instrumentation

**Figure 1:**
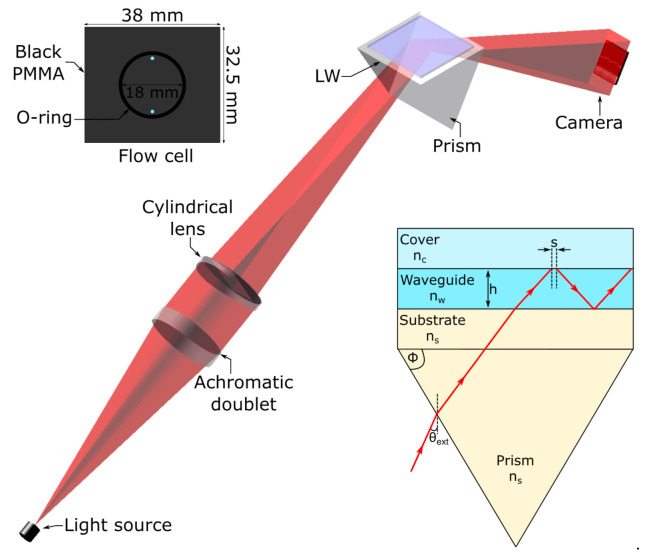
Schematic of the LW instrumentation (the base of the prism is 30 mm by 30 mm) where the insets show the flow cell used in this work, and a ray diagram showing partial confinement of light in LWs (where θ_ext_ is the external angle of incidence, s is the Goos–Hänchen shift, and n_c_, n_w_, n_s_ are the refractive indices of cover/sample, waveguide and substrate respectively. For LWs, n_c_ < n_w_ < n_s_).

As shown in Figure 1, light from a point source red LED (TL-6, iC-Haus, 640 nm) was passed through optical lenses (40 mm focal length achromatic doublet and 63 mm focal length cylindrical lens) to illuminate the LW with a wedge-shaped light beam. The LW was mounted on a BK7 equilateral prism (Qioptic Photonics, Denbighshire, UK) using a RI matching oil and the reflected light was captured using a 20 Mpixel CMOS camera (MER-2000–19U3M-L, Daheng Imaging, Beijing, China). A single channel flow cell was made from 3 mm thick poly(methyl methacrylate) (PMMA) and the dimensions of the channel are provided in the inset in Figure 1. The flow cell was mounted on the top of the LW and held in place using a clamping plate connected to a temperature-controlled recirculating water bath. The solutions used for testing the chitosan LWs were pumped through the flow cell using a peristaltic pump (Minipuls^®^ 3, Gilson, Bedfordshire, UK) at a flow rate of 200 µL/min. The RI of the solutions were measured using RFM900-T refractometer (Bellingham and Stanley, Kent, UK) with an accuracy of ±1 × 10^−5^ RIU.

## 3. Results and Discussion

### 3.1. Optimisation of the Diffraction-Based LW

**Figure 2:**
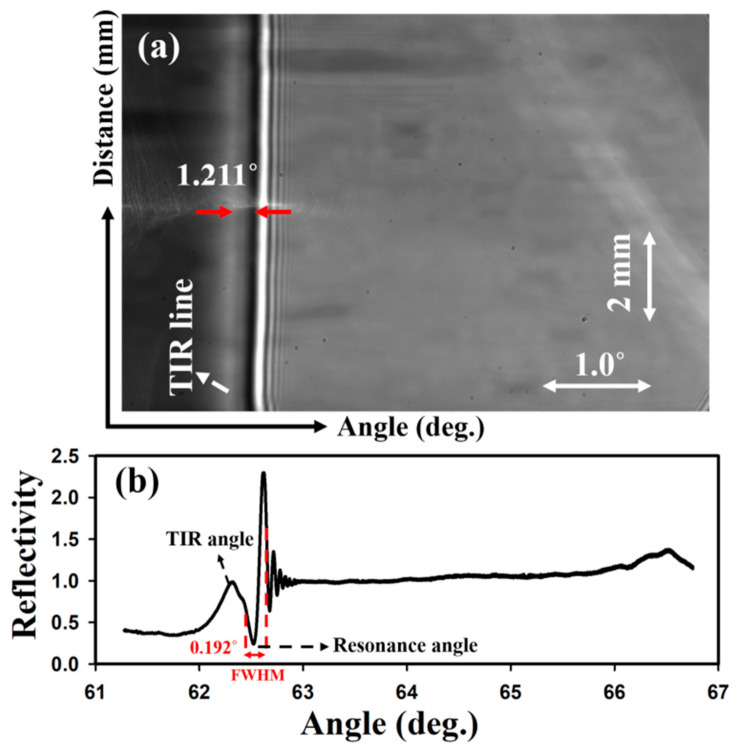
(**a**) Output of a chitosan LW with flow cell mounted on the top and filled with HEPES buffer, and (**b**) the corresponding reflectivity curve (where angle is θ_ext_ as marked in Figure 1).

A typical output of a diffraction-based LW with an unpatterned chitosan film is shown in Figure 2a where interference fringes were observed at the resonance angle (θ_R_). The θ_R_ was ~1.211° away from the total internal reflection (TIR) angle. The corresponding reflectivity curve is provided in Figure 2b and the FWHM of the dip-peak pair was ~0.192°. As discussed in our previous work [12], for LWs where the RI between the waveguide and sample solution is <0.01, exponentially decaying interference fringes are observed at resonance angles. The reflectivity curve was normalised to the intensity between 63.5 and 65° because the reflectivity in this region is expected to be unity. The reflectivity of the interference peak is greater than one because of constructive interference, while the reflectivity of the interference dip is less than one because of destructive interference. At angles greater than the resonance angles, the reflectivity is one because of TIR. The small hump in the grey-scale values observed between ~66 and 67° in Figure 2b is a result of the stray reflections that were observed as a white streak in Figure 2a.

A simplex optimisation was performed to extract the thickness and RI of the chitosan LWs from the experimental reflectivity curves. The typical thickness and RI of the chitosan films were estimated to be 1.95 µm and 1.3437−1.28 × 10^−4^i respectively. The imaginary RI of the chitosan films indicates that there may be some scattering losses. The RIS of the chitosan LW was 121.09 ± 4.30° RIU^−1^ (see Figure S1 in Supplementary Materials for details). The FOM was 630.68 ± 22.40 RIU^−1^, which is ~1.8 times lower than poly(acrylamide) LWs [12], and may be because of higher scattering losses in the chitosan than poly(acrylamide) films. In all cases, the error bars represent area-to-area variability across the width of the flow cell mounted on the LW. This area-to-area variability across the sensor surface arose from the variations in the waveguide thickness resulting from the spin coating process. Within an experimental run using a single device, the primary factor determining the measurement noise is the pressure pulsation introduced by the peristaltic pump used to drive solutions into the flow cell. The device-to-device reproducibility is primarily determined by the waveguide material, which, in this case, is a natural material (chitosan) and has inherent variability that is difficult to quantify.

Figure S2 in Supplementary Materials shows the response for glycerol (92 D) and PEG (10, 100 and 300 kD) solutions of 10 mg/mL. It is observed that the shift in the resonance angle for glycerol is 0.16 ± 0.01° while for 10, 100 and 300 kD PEG solutions is 0.14 ± 0.01°, 0.134 ± 0.01°, and 0.13 ± 0.01°, respectively. The RI values of each solution are also provided in Figures S1 and S2 in Supplementary Materials. The RIS for each solution was estimated by taking a ratio of the shift in resonance angle and the RI of the solution. Subsequently, the porosity was estimated using Equation (1) [13]:

(1) $$P=\frac{RIS_{p}-RIS_{ew}}{RIS_{gly}-RIS_{ew}}\times 100\%$$

where *RIS_p_* is the refractive index sensitivity for a given PEG solution, *RIS_ew_* is the sensitivity to refractive index in the evanescent field and *RIS_gly_* is the sensitivity to glycerol. *RIS_ew_* is determined by modelling using the thickness and refractive index of the waveguide. Based on diffusion studies (see Figure S2 in Supplementary Materials for details) and Equation (1), the chitosan films had a porosity relative to glycerol of 93%, 81% and 76% for 10 mg/mL solutions of PEGs of molecular weight 10, 100 and 300 kD.

**Figure 3:**
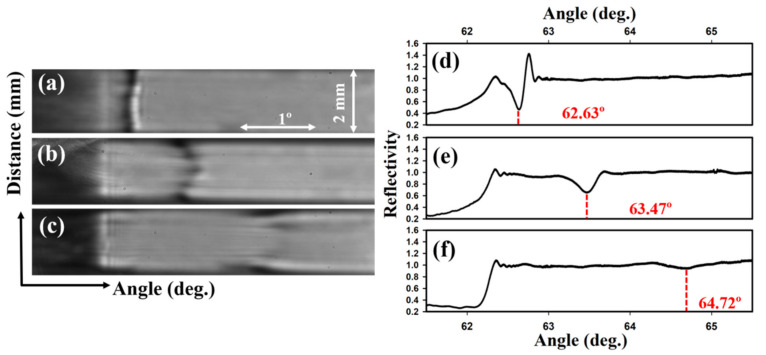
2D and the corresponding 1D reflectivity of LWs after streptavidin immobilisation to chitosan films activated with (**a**,**d**) 0 mg/mL, (**b**,**e**) 1 mg/mL and (**c**,**f**) 2 mg/mL of PC-biotin (where angle is θ_ext_ as marked in Figure 1).

Next, we optimised the concentration of the PC-biotin solution used to treat the chitosan films. Figure 3 shows that as the concentration of PC-biotin solution was increased, larger amounts of streptavidin were immobilised in the waveguide, which was reflected as a bigger shift of the resonance angle (Δθ_R_) of the LWs. The angle shifts of 0.44 ± 0.01°, 0.75 ± 0.02° and 1.93 ± 0.02° for 0.47, 1, and 2 mg/mL PC-biotin respectively correspond to streptavidin concentrations (*c_strep_*) of 0.35 ± 0.01, 0.59 ± 0.02 and 1.53 ± 0.02 mM respectively based on the RIS of the LW sensor, a RI increment (*δn/δc*) of 0.19 g^−1^ mL of typical proteins [29] and using Equation (2) where *M_strep_* is molecular weight of streptavidin.

(2) $$c_{strep}=\frac{\Delta \theta_{R}}{M_{strep}\times RIS\times \left(\delta n/\delta c\right)}$$

The concentration of amines (*c_amines_*) in chitosan is given by Equation (3).

(3) $$c_{amines}=\frac{c_{chitosan}}{D\times M_{g}+\left(1-D\right)\times M_{ag}}$$

where *c_chitosan_* is 10 g/L i.e., concentration of chitosan solution used to make the films, *D* is degree of deacetylation (90%), *M_g_* is the molecular mass of a glucosamine unit and *M_ag_* is the molecular mass of an acetylglucosamine unit. The concentration of amines in the film was estimated to be ~60 mM. The ratio of *c_strep_* and *c_amines_* was then calculated, and only a maximum of 2.5% of the available amines in chitosan were occupied by streptavidin. However, as the concentration of PC-biotin solution was increased to 2 mg/mL, a broad and shallow dip in reflectivity was observed at the resonance angle of the LWs after streptavidin binding (see Figure 3c,f). This can be explained by considering that the streptavidin binding increased the RI of the chitosan waveguide. As the RI of the waveguide increases, the fraction of light that leaks at the waveguide/substrate interface increases, which means that the optical losses in the waveguide increase. These increased losses cause the dip-peak pair at the resonance angle to be replaced by a broad shallow dip, which is quite hard to track to monitor changes in the resonance angle for sensing. This shallow dip is consistent with our previous work [12], which showed that increasing the RI of the waveguide resulted in a decrease in the amplitude of the interference fringes, which eventually disappeared when the RI contrast between the waveguide and sample exceeded 0.01.

To create active and inactive regions for analyte binding in the LW, a photocleavable biotin reagent, 1 mg/mL PC-biotin solution was used. It comprises a PEG spacer arm with a nitrobenzyl photocleavable moiety linked to an amine reactive N-hydroxysuccinimidyl (NHS) group at one end, and a biotin moiety at the other end. When the chitosan film was treated with PC-biotin, the NHS region reacted specifically with the primary amines (-NH_2_) of chitosan. Streptavidin was then bound to the biotin end of the photocleavable linker. Illumination with near-UV light cleaved the linker, removing the streptavidin from the exposed area, allowing selective immobilisation of biotinylated species (i.e., protein A–biotin, PAB) in the next step.

**Figure 4:**
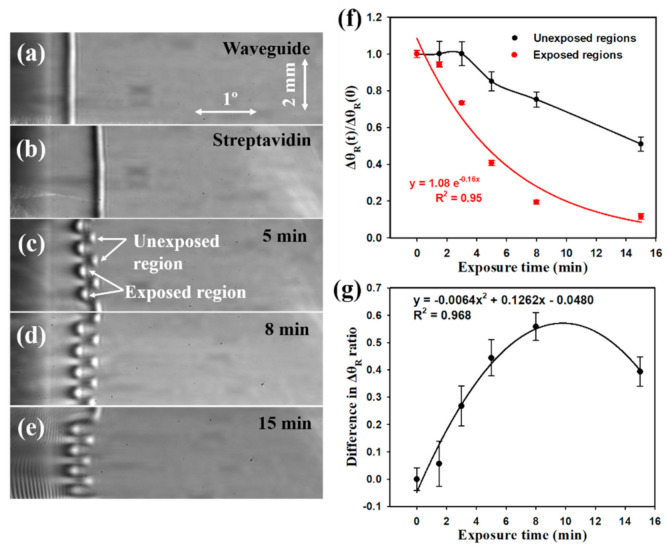
Output of a diffraction-based LW (**a**) before and (**b**) after streptavidin immobilisation, and its selective removal by exposure to 365 nm light for (**c**) 5 min, (**d**) 8 min and (**e**) 15 min, (**f**) ratio and (**g**) absolute difference of Δθ_R_ after and before photo exposure *versus* exposure time.

Figure 4 shows the output image of a chitosan LW (Figure 4a), the LW after immobilisation of streptavidin (Figure 4b), and the photopatterned LW (Figure 4c–e), with alternating active (presence of streptavidin) and inactive (absence of streptavidin) regions for exposure times of 5, 8, and 15 min, respectively. Δ*θ_R_*(0) and Δ*θ_R_*(*t*) in Figure 4f,g are defined in Equations (4) and (5), respectively.

(4) $$\Delta \theta_{R}\left(0\right)=\theta_{strep}-\theta_{wg}$$

(5) $$\mathrm{And}\;\Delta \theta_{R}\left(t\right)=\theta_{t}-\theta_{wg}$$

where *θ_wg_* is the resonance angle before streptavidin immobilisation (shown in Figure 4a), *θ_strep_* is the resonance angle after streptavidin immobilisation (shown in Figure 4b) and *θ_t_* is the resonance angle after exposure to 365 nm light for time ‘t’ (shown in Figure 4c–e).

As shown in Figure 4f, the Δ*θ_R_*(*t*)/Δ*θ_R_*(0) for the exposed regions decreased exponentially as the exposure time was increased. The unexposed region was expected to show a slower exponential decay, but the relatively large error bars make it difficult to determine the relationship between Δ*θ_R_* and exposure time. The exposure time of 1.5 min released ~6% of streptavidin and a maximum of ~88% was released for exposure time of 15 min. Although the exposure time of 15 min removed a significant fraction of streptavidin (~88%) from the exposed regions of the waveguide, it also removed up to ~49% of streptavidin from the unexposed area, leaving fewer active binding sites for the sensing of protein A–biotin (PAB) and IgG. A plot of the difference in the ratios of Δθ_R_ from unexposed regions to exposed regions against exposure time (see Figure 4g) followed a parabolic profile with a peak at the exposure time of 9.9 min, which is close to the experimental value of 8 min. For this reason, 8 min exposure was used in the subsequent work.

There is no upper limit on the grating period, but the lower bound on the grating period was limited by the ability to visualise the resonance angle corresponding to the individual strips. As can be seen in Figure 4c,d, the higher RI of the unexposed strips causes additional diffraction at the strip edges, which means that the resonance angle of the individual strips become harder to resolve as the strip width decreases. A grating period of 2 mm was found to be optimum.

### 3.2. Self-Referenced Diffraction-Based LW Biosensor

**Figure 5:**
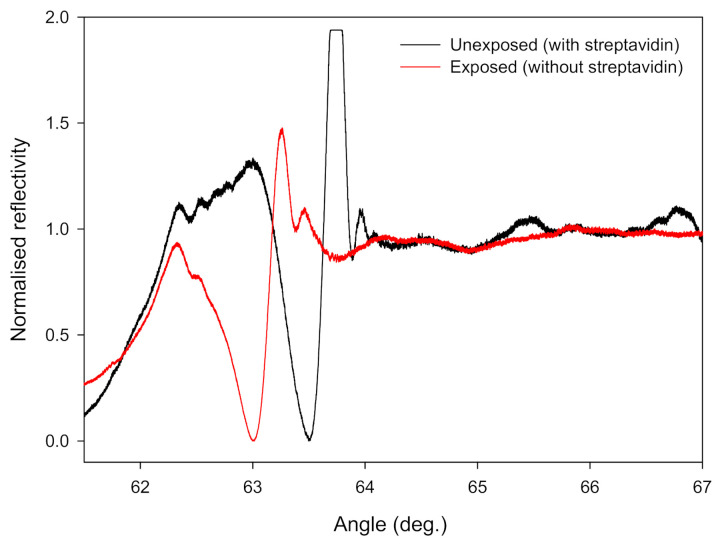
Normalised reflectivity curves of the regions of diffraction-based LWs with and without immobilised streptavidin (where angle is θ_ext_ as marked in Figure 1).

The reflectivity profiles of a diffraction-based LW for regions unexposed and exposed (i.e., with high and low streptavidin loading, respectively) to 365 nm light are shown in Figure 5. Figure 5 clearly shows that the resonance angles corresponding to the LW with high and low streptavidin loading are different. The feasibility of a diffraction-based LW with alternating regions of high and low streptavidin loading for referencing was investigated next. To do this, absolute and differential shifts in the resonance angles of high and low streptavidin loading (i.e., sensor and reference) regions to common-mode effects (i.e., temperature, sample composition caused by non-adsorbing and adsorbing species) and analyte binding were determined, and the results are discussed below.

#### 3.2.1. Compensation for Changes in Temperature

**Figure 6:**
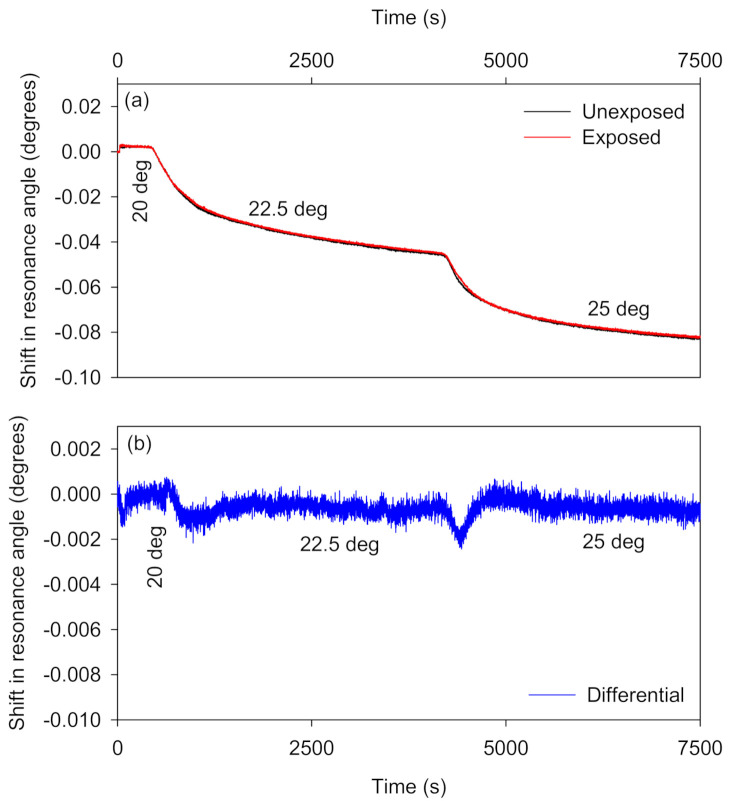
(**a**) Absolute and (**b**) differential shifts in the resonance angles of the self-referenced diffraction-based LW when the system temperature was varied.

The temperature of the water bath used to recirculate the fluid through the plate mounted on top of the flow cell and LW assembly was increased from 20 °C to 25 °C in steps of 2.5 °C. As shown in Figure 6a, the absolute shifts in resonance angles of sensor and reference regions decrease by ~0.08° as the temperature increases from 20 °C to 25 °C. Figure 6a also shows that the shifts in the resonance angles of the sensor and reference regions because of temperature changes were approximately equal. The differential signal produced by this temperature change was ~98% lower than the absolute shifts in the resonance angles of the sensor and reference regions. Thus, differential measurements between the shifts in the resonance angles of the sensor and reference regions can greatly reduce the temperature effect from the overall LW output. The unexposed regions seem to respond slightly less rapidly to changes in temperature than the exposed regions. It seems unlikely that this is because of differences in the thermal diffusivity or heat capacity of the very thin chitosan layer, so might be a result of physical changes in the hydrogel because of the difference in water content.

#### 3.2.2. Compensation for Changes in Sample Composition

**Figure 7:**
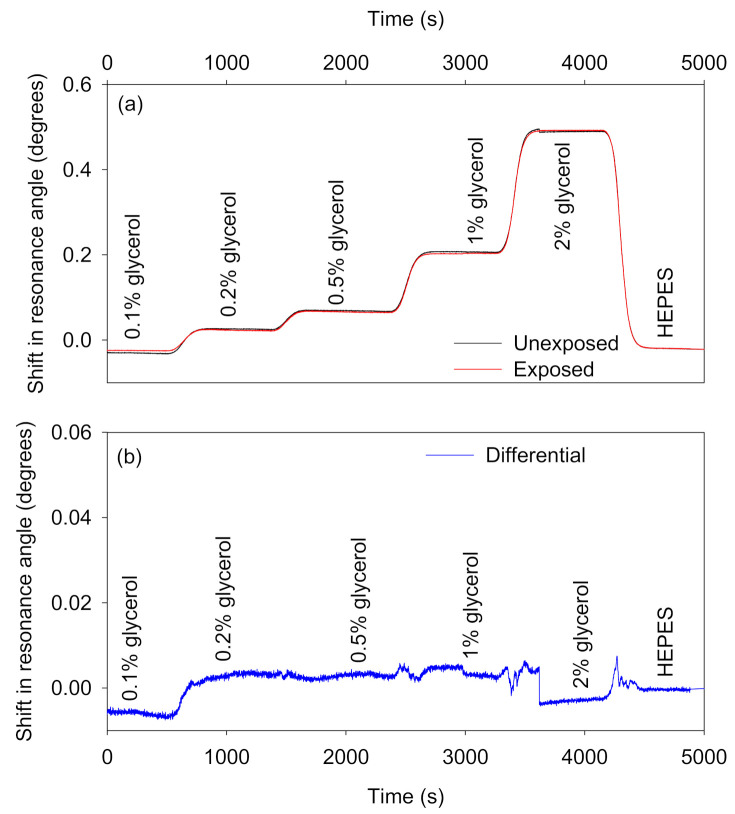
(**a**) Absolute and (**b**) differential shifts in the resonance angles of the self-referenced diffraction-based LW to changes in sample composition caused by different concentrations of glycerol solutions.

**Figure 8:**
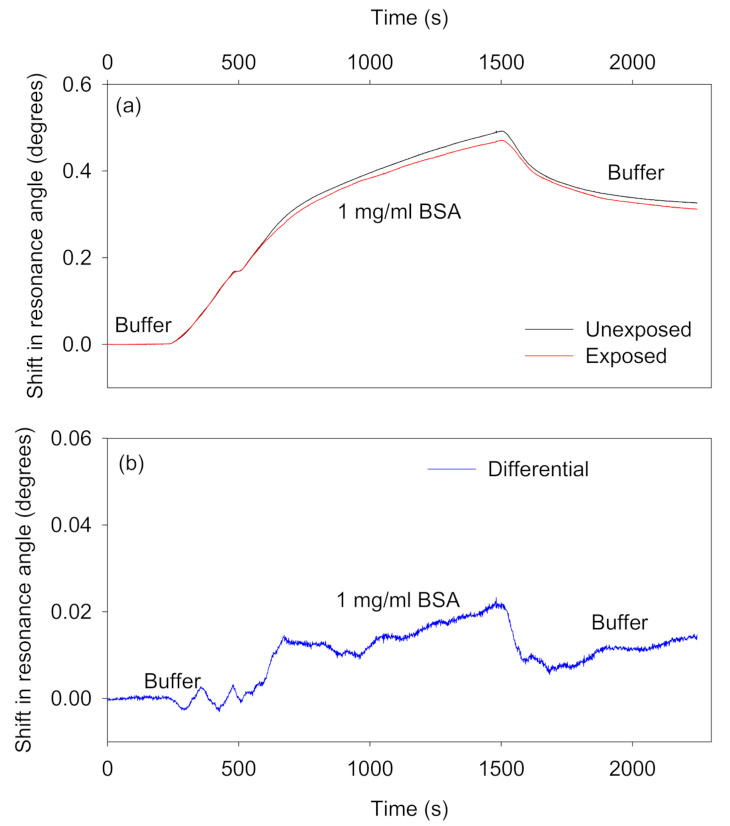
(**a**) Absolute and (**b**) differential shifts in the resonance angles of the self-referenced diffraction-based LW to non-specific adsorption of 1 mg/mL BSA prepared in 100 mM HEPES buffer, pH 7.4.

- Non-adsorbing interferents: Figure 7 shows the absolute and differential shifts in resonance angles as a result of changes in RI caused by different concentrations of glycerol solutions. The RI of glycerol solutions of different concentrations are provided in Table S1 in Supplementary Materials. On average, the differential response because of changes in bulk RI was ~99% lower than the absolute shifts in the resonance angles of the sensor and reference regions.
- Adsorbing interferents: Ideally, species in samples should not non-specifically adsorb to the waveguide materials. Alternatively, the non-specific adsorption of species to sensor and reference regions should at least be similar so that differential measurements can be performed to remove the contribution of non-specific adsorption from the overall output of the biosensor. We investigated the potential of the reported self-referenced LW to eliminate the effect of non-specific adsorption by using 1 mg/mL BSA solution prepared in 100 mM HEPES buffer, pH 7.4. As shown in Figure 8a, the resonance angle of sensor and reference regions increased as BSA solution was introduced on top of the patterned chitosan LW. The resonance angles did not return back to baseline after a buffer wash. This may be a result of electrostatic interactions between BSA and the chitosan films as well as hydrophobic interactions between BSA and the NVOC in unexposed regions of the film. The isoelectric point of BSA is 4.7–5.1 [30], whereas the pK_a_ of the amines in chitosan is about 6.5 [31], which means that BSA will be negatively charged at the selected pH of the buffer, and hence can interact with positively charged primary amines in chitosan films [32]. Since the difference in response to BSA between the exposed and unexposed regions is quite small, it seems likely that electrostatic interactions are more significant. The shifts in resonance angles of sensor and reference regions because of non-specific adsorption of BSA were, however, similar. As a result, as shown in Figure 8b, the differential signal was up to 97% lower than the absolute shifts in the resonance angles of the sensor and reference regions. The remaining small difference between the exposed and unexposed regions can be explained by non-specific interaction of BSA with the higher concentration of streptavidin in the unexposed regions.

#### 3.2.3. Kinetic Analysis of Analyte Binding

The difference in the concentration of streptavidin in the exposed and unexposed regions will make some difference to the kinetics of analyte binding, for two reasons. Firstly, removal of streptavidin will make a difference to the porosity of the film, changing the rate at which other molecules can diffuse into the film and bind to the remaining streptavidin. Secondly, we can assume a laminar flow regime with a Nernstian diffusion layer, based on the calculated Reynolds number of 0.74 for the flow cell and flow velocity used. In this case, the surface concentration of binding sites will make a difference to the rate at which analyte can bind. The expression for the time dependence of the surface concentration of bound analyte is given by Equation (6) [33], where *D* is the analyte diffusion coefficient, *K* the dissociation constant for the binding pair, *t* is time, *δ* is the thickness of the diffusion layer and *Γ_lim_* the surface concentration of streptavidin.

(6) $$\frac{\Gamma_{t}}{\Gamma_{\mathrm{eq}}}=1-e^{-\frac{DKt}{\delta \Gamma_{lim}}}$$

*D* is a combination of the diffusion coefficient of analyte in free solution and in the hydrogel. Fitting the observed values of peak angle shift for PAB to immobilised streptavidin to an exponential rise gives values for the exponent of 4.86 × 10^−3^ s^−1^ (r = 0.9903) and 1.74 × 10^−3^ s^−1^ (r = 0.9793) for the unexposed and exposed regions, respectively. The ratio of these two values was 2.79, while the ratio of *Γ_lim_* in the unexposed and exposed regions was 3.88, which implies that one of the other parameters in the expression for the exponential term also differed between the exposed and unexposed regions. Since the dissociation constant *K* is not expected to be dependent on the (low) density of the binding sites and *δ* is dependent only on the dimensions of the flow cell and flow rate, which were the same for all measurements, the only remaining parameter that could vary is the diffusion coefficient *D*. The ratio of diffusion coefficients for PAB in the exposed and unexposed areas is 0.719. This means that the exposed areas are less porous, even though more streptavidin has been removed.

Fitting of the IgG binding to immobilised PAB to an exponential rise gave a value of the exponent of 9.05 × 10^−4^ s^−1^ (r = 0.9991) for the exposed regions and 6.83 × 10^−4^ s^−1^ (r = 0.9990) for the unexposed regions. This gives a ratio of the diffusion coefficients in the exposed and unexposed areas of 0.34, which is consistent with the larger size of IgG compared to PAB if the exposed areas are less porous than the unexposed areas.

#### 3.2.4. Temperature Compensation during Analyte Unbinding

**Figure 9:**
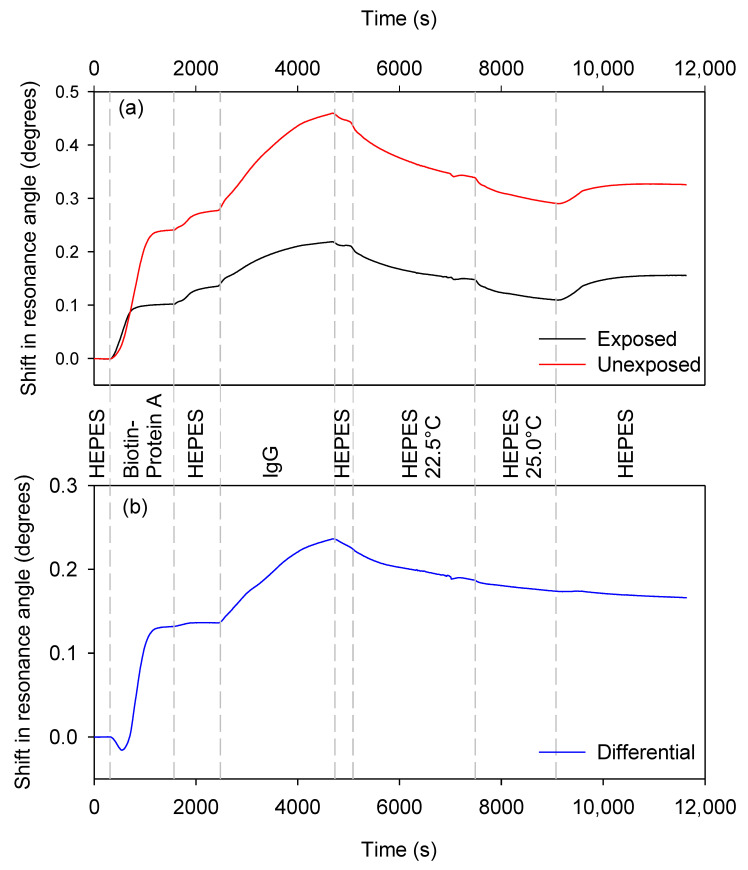
(**a**) Absolute and (**b**) differential shifts in the resonance angles of the self-referenced diffraction-based LW to protein A–biotin (PAB) (labelled as biotin-Protein A) and IgG binding and temperature changes during HEPES washing.

To show that the effect of temperature changes could be significantly suppressed while performing analyte binding, a chitosan sensor was prepared and maintained at 20 °C while PAB was pumped through the flow cell. After a HEPES wash, IgG was allowed to bind to the immobilised PAB, after which a further brief HEPES wash was performed. The temperature of the flow cell was then raised successively to 22.5 °C and 25.0 °C and then returned to 20 °C while HEPES was being pumped through the flow cell. Figure 9 shows the resulting sensorgrams, where (a) is a plot of the absolute resonance angle shifts averaged over three strips of the unexposed (sensing) and exposed (reference) waveguide and (b) is the average difference between the unexposed and exposed areas. Changes in the absolute response are clearly visible as the temperature was changed, but have been significantly reduced in the differential plot. Fitting the data for the HEPES wash with temperature changes to an exponential decay gives an RMS residual error of 0.001382° for the differential plot and 0.0126° for the unexposed (sensing) regions. This is a factor of 9 reduction in the effect of temperature. This reduction in sensitivity to temperature, however, does come with a reduction in sensitivity to about half that of the absolute sensitivity of the unexposed regions.

Table 2 gives a comparison between our previous work on metal-clad leaky waveguides (MCLWs) comprising stacked agarose waveguide layers and this work on diffraction-based LWs comprising chitosan waveguides in a side-by-side self-referencing configuration. In all cases the present work provides better cancellation of common-mode effects, and provides considerably better differential sensitivity to analyte binding. This is because of the significant overlap between the two modes in the stacked configuration, which does not occur with the side-by-side configuration. The reduction in differential sensitivity in this work is a result of the incomplete removal of streptavidin in the exposed regions and its partial removal in the unexposed regions.

## 4. Conclusions

Referencing, where a plurality of independent measurements is performed to separate common-mode effects (i.e., changes in temperature, wavelength, and sample composition) from analyte binding, is of paramount importance for practical applications of label-free (optical) biosensors. In this paper, a novel combination of a self-referenced diffraction-based optical leaky waveguide (LW) biosensor with an array of interspersed sensor and reference strips and a pitch of ~1 mm was fabricated using a photocleavable biotin linker immobilised in chitosan films. The absolute refractive index sensitivity and figure of merit of the LW sensor were 121.09 ± 4.30° RIU^−1^ and 630.68 ± 22.40 RIU^−1^, respectively. We demonstrated that differential measurements between the sensor and reference regions reduced the effect of changes in temperature over 5 °C and sample composition because of non-adsorbing and adsorbing species by ~98%, ~99%, and ~97%, respectively compared to the absolute measurements. The differential sensitivity to analyte binding was reduced to 49% of the absolute sensitivity, but this decrease in sensitivity was far outweighed by the benefits of compensation for changes in temperature and sample composition. LWs are largely non-dispersive, and hence changes in wavelength have an insignificant effect on their output. Future work will focus on investigating the use of synthetic hydrogels, which offer lower scattering, better control over the density of functional groups, higher batch-to-batch reproducibility, and possibilities for covalent attachment to substrates, for making such self-referenced LW biosensors.

## Figures and Tables

**Table 1:** Comparison of the theoretical dispersion characteristics of surface plasmon resonance (SPR), resonant mirror (RM) and diffraction-based leaky waveguides (LWs).

Device	FWHM (°)	Sensitivity (° nm^−1^)	DFOM (nm)
SPR (TM)	4.3185	0.0657	65.8
RM (TM)	1.0212	8.62 × 10^−3^	118.5
RM (TE)	0.1941	0.0174	11.16
Diffraction-based LW (TM)	0.0518	3.62 × 10^−4^	143.1
Diffraction-based LW (TE)	0.0476	1.60 × 10^−4^	296.7

**Table 2:** Comparison of our previous work using stacked metal-clad leaky waveguide (MCLW) layers and this work using side-by-side sensing and reference strips.

Device	Compensation for Change in Temperature	Compensation for Changes in Sample Composition		Reduction in Sensitivity to Analyte Binding
		Non-Adsorbing Interferents	Adsorbing Interferents	
Stacked MCLW [34]	94%	97%	Not tested	78%
This work	98%	99%	97%	49%

## Supplementary Materials

The following are available online at https://www.mdpi.com/2079-6374/10/10/134/s1, Figure S1: Sensorgram of a chitosan LW irrigated with glycerol solutions prepared in 100 mM HEPES, pH 7.4 at different times where the inset plots shift in resonance angle *versus* the refractive index of glycerol solutions, Figure S2: Shift in resonance angle of the LW for glycerol and PEG (35, 100 and 300 kD) solutions of 10 mg/mL concentration prepared in 100 mM HEPES, pH 7.4, Table S1: RI of glycerol solutions of different concentrations measured using RFM900-T refractometer (Bellingham and Stanley, Kent, UK) with an accuracy of ±1 × 10^−5^.
