# Supplementary material for: A Self-Referenced Diffraction-Based Optical Leaky Waveguide Biosensor Using Photofunctionalised Hydrogels
Source: Biosensors (Basel). 2020 Sep 24;10(10):134. doi: 10.3390/bios10100134 (PMC7601400; doi:10.3390/bios10100134)
Supplement: Supplementary file 1 [file biosensors-10-00134-s001.pdf]

Article

A Self-Referenced Diffraction-Based Optical Leaky Waveguide Biosensor Using Photofunctionalised Hydrogels

Anil K. Pal [1], Nicholas J. Goddard [2,*], Hazel J. Dixon [1] and Ruchi Gupta [1,*]

[1] School of Chemistry, University of Birmingham, Birmingham B15 2TT, UK; a.k.pal@bham.ac.uk (A.K.P.); hjd839@student.bham.ac.uk (H.J.D.)

[2] Process Instruments (UK) Ltd, Burnley, BB12 0BT, UK.

* Correspondence: nick.goddard@processinstruments.net (N.J.G); r.gupta.3@bham.ac.uk (R.G.)

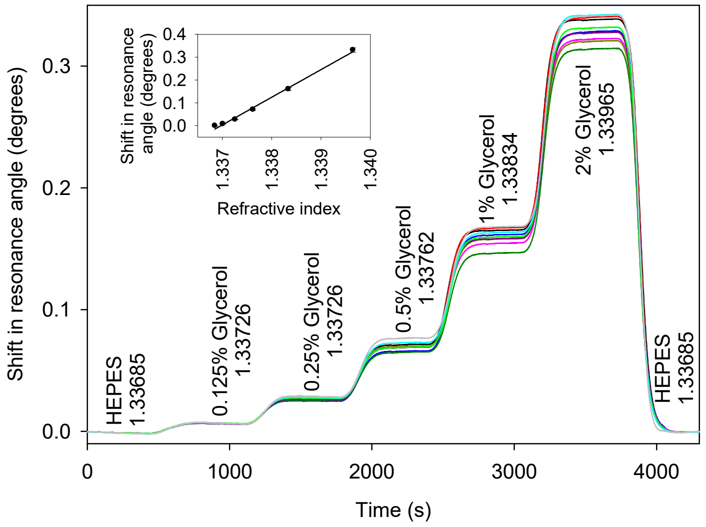

Figure S1. Sensorgram of a chitosan LW irrigated with glycerol solutions prepared in 100 mM HEPES, pH 7.4 at different times where the inset plots shift in resonance angle versus the refractive index of glycerol solutions.

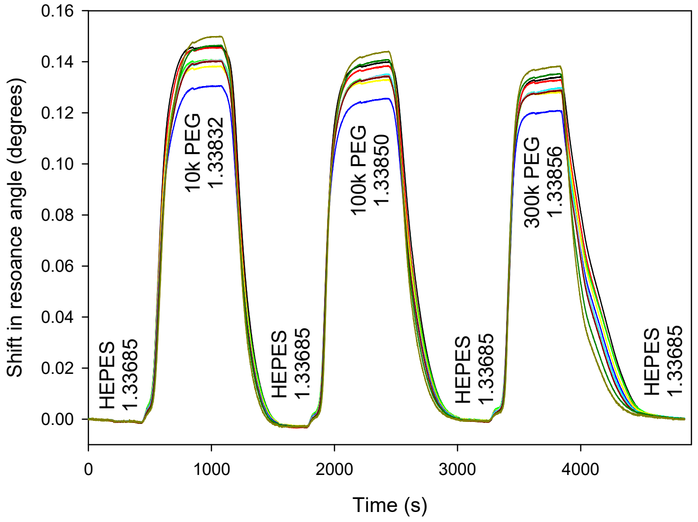

Figure S2. Shift in resonance angle of the LW for glycerol and PEG (35, 100 and 300 kD) solutions of 10 mg/ml concentration prepared in 100 mM HEPES, pH 7.4.

Table S1. RI of glycerol solutions of different concentrations measured using RFM900-T refractometer (Bellingham and Stanley, Kent, UK) with an accuracy of $\pm 1 \times 10^{-5}$.

Concentration of glycerol (%, w:v)	Refractive index (RI)
Buffer i.e. 0	1.33698
0.1	1.33711
0.2	1.33727
0.5	1.33774
1	1.33845
2	1.33992
